# Coagulation Factor X Regulated by CASC2c Recruited Macrophages and Induced M2 Polarization in Glioblastoma Multiforme

**DOI:** 10.3389/fimmu.2018.01557

**Published:** 2018-07-06

**Authors:** Yan Zhang, Jianbo Feng, Haijuan Fu, Changhong Liu, Zhibin Yu, Yingnan Sun, Xiaoling She, Peiyao Li, Chunhua Zhao, Yang Liu, Tao Liu, Qiang Liu, Qing Liu, Guiyuan Li, Minghua Wu

**Affiliations:** ^1^Hunan Provincial Tumor Hospital and the Affiliated Tumor Hospital of Xiangya Medical School, Central South University, Changsha, China; ^2^The Key Laboratory of Carcinogenesis of the Chinese Ministry of Health, The Key Laboratory of Carcinogenesis and Cancer Invasion of the Chinese Ministry of Education, Cancer Research Institute, Central South University, Changsha, China; ^3^The Second Xiangya Hospital, Central South University, Changsha, China; ^4^The Third Xiangya Hospital, Central South University, Changsha, China; ^5^The Xiangya Hospital, Central South University, Changsha, China

**Keywords:** tumor-associated macrophages, polarization, glioblastoma multiforme, extracellular signal-related kinase 1/2, AKT

## Abstract

Tumor-associated macrophages (TAMs) constitute a major component of inflammatory cells in the glioblastoma multiforme (GBM) tumor microenvironment. TAMs have been implicated in GBM angiogenesis, invasion, local tumor recurrence, and immunosuppression. Coagulation factor X (FX) is a vitamin K-dependent plasma protein that plays a role in the regulation of blood coagulation. In this study, we first found that FX was highly expressed and positively correlated with TAM density in human GBM. FX exhibited a potent chemotactic capacity to recruit macrophages and promoted macrophages toward M2 subtype polarization, accelerating GBM growth. FX bound to extracellular signal-related kinase (ERK)1/2 and inhibited p-ERK1/2 in GBM cells. FX was secreted in the tumor microenvironment and increased the phosphorylation and activation of ERK1/2 and AKT in macrophages, which may have been responsible for the M2 subtype macrophage polarization. Moreover, although the lncRNA CASC2c has been verified to function as a miR-101 competing endogenous RNA (ceRNA) to promote miR-101 target genes in GBM cells, we first confirmed that CASC2c did not function as a miR-338-3p ceRNA to promote FX expression, and that FX was a target gene of miR-338-3p. CASC2c interacted with and reciprocally repressed miR-338-3p. Both CASC2c and miR-388-3p bound to FX and commonly inhibited its expression and secretion. CASC2c repressed M2 subtype macrophage polarization. Taken together, our findings revealed a novel mechanism highlighting CASC2c and FX as potential therapeutic targets to improve GBM patients by altering the GBM microenvironment.

## Introduction

Glioblastoma multiforme (GBM) is the most common type of primary brain tumor in adults and is associated with poor prognosis (1, 2). One challenge of GBM is genetic heterogeneity, while noncancerous stromal cells in the tumor microenvironment are genetically stable as therapeutic targets (3). Tumor-associated macrophages (TAMs) in particular are associated with high tumor grades and poor prognosis in many cancers, including GBM (4). Abundant macrophages infiltrate GBM and promote tumor progression in multiple aspects. TAMs secrete cytokines, including interleukin-6 (IL-6), interleukin-10 (IL-10), tumor necrosis factor-α (TNF-α), and interferon-γ, which have been shown to promote tumor cell growth (5). TAMs could also facilitate angiogenesis by releasing vascular endothelial growth factor-α and associating with adjacent endothelial tip cells, facilitating vascular anastomosis (6). A population of TIE2^+^ macrophages also promotes tumor cell intravasation into circulation by alignment along the vessels (7). TAMs also secrete various cytokines and chemokines that suppress CD4^+^ and CD8^+^ T cell effector function directly or indirectly by recruiting regulatory T cells to the tumor microenvironment (8).

Macrophages are categorized into classically activated (M1) and alternatively activated (M2) subtypes based on their polarization status (9). M1 macrophages represent tumor-suppressive macrophages and participate in polarized T helper 1 (Th1) responses, *via* the production of the pro-inflammatory mediators TNF-α, IL-1β, and IL-6 (10). M1 macrophages also have antigen presentation capacity by upregulating cell surface molecules MHC II and costimulatory molecules CD80 and CD86 (11). Conversely, M2 macrophages represent tumor-supportive macrophages and participate in polarized Th2 immune responses, *via* secreting immunosuppressive cytokines IL-10 and TGF-β, downregulation of antigen-presenting molecules, including MHC II, CD80, and CD86, and decreasing phagocytic capacity (12). Cell markers on M2 macrophages include CD163, ARG1, HMOX1, LYVE1, MRC1, SerpinB2, and STAB1, whereas CD11c, IL-1β, IL-12α, CXCL9, IL-12β, CXCL10, and iNOS have been suggested as markers of M1 macrophages (12, 13). TAMs in GBM tumors express strong M2 markers (14). The correlation between tumor grades and the M2 TAM abundance further suggests that M2 TAMs play an important role in GBM progression.

Coagulation factor X (FX) is a vitamin K-dependent plasma protein known to be an important player in the regulation of blood coagulation by converting prothrombin into thrombin (15). Activated FX (FXa) occupies a central position in the coagulation cascade and plays a role in tissue remodeling, fibrosis, and cancer *via* activating protease-activated receptors (PAR)-1 or PAR-2 to mediate intracellular signaling (16, 17). Classically, FXa-induced PAR signaling induces phosphoinositide hydrolysis, leading to calcium oscillation. FXa also triggers the phosphorylation of mitogen-activated protein kinases (MAPKs), specifically extracellular signal-related kinase (ERK) and c-Jun N-terminal kinase, activates the PI3K–AKT/PKB pathway and the phosphorylation of mTOR, leading to cell proliferation, differentiation, and migration (18). Furthermore, FXa regulates inflammatory signaling by inducing the expression of IL-6, IL-8, monocyte chemotactic protein-1, and intracellular adhesion molecule (19). Many observations have shown ectopic expression of FX in cancer cells, including ovarian cancer, small lung cell carcinoma, renal cell carcinoma, and malignant melanoma (20). Our previous studies have indicated that FX overexpression in glioma was due to promoter hypomethylation, and its protein expression correlated with tumor grade and overall survival (21).

In this study, we demonstrated that FX had chemotactic ability that recruited macrophages in GBM and mainly promoted macrophage polarization to M2 subtype, facilitating tumor growth. Furthermore, FX interacted with ERK1/2 and decreased p-ERK1/2 in GBM cells, while it was secreted into the tumor microenvironment and increased p-ERK1/2 and p-AKT in macrophages, which played a role in macrophage polarization.

## Materials and Methods

### Cell Culture

The human astrocytoma cell line U251 and mouse glioma cell line GL261 were purchased from cell banks of the Chinese Academy of Sciences (Shanghai, China). The normal human astrocyte cell line HEB was obtained from the Guangzhou Institute of Biomedicine and Health, Chinese Academy of Sciences (Guangzhou, China) (22). Primary cultured GBM cells (G1124, G1104) (23) were separated from human GBM samples by the Department of Neurosurgery, Xiangya Hospital, Central South University. All cells were cultured in Dulbecco’s modified Eagle’s medium (DMEM, HyClone) supplemented with 10% fetal bovine serum (FBS, Biological Industries) and 1% penicillin/streptomycin (HyClone) at 37°C and 5% CO_2_ in a humidified atmosphere.

### Patients and Tissue Samples

The human astrocytoma tissue samples were acquired from the Department of Neurosurgery, Xiangya Hospital, Central South University with informed consent of the patients, which was approved by the Joint Ethics Committee of the Central South University Health Authority. Paraffin sections of 4-µm thickness were produced according to the manufacturing process for HE and immunohistochemical staining. Frozen sections of 8-µm thickness were made according to standard procedure for immunofluorescence staining.

### Plasmids

Factor X was amplified from G1124 cells and cloned into plasmids pEGFP-C1, p3xFLAG-CMV-10, and pcDNA3.1. ERK1 and ERK2 were cloned from 293 cells and fused into pDsRed1-N1 plasmid. The 3′UTR regions of FX and CASC2c were synthesized by Sangon Biotech Company and inserted into a pmirGLO Vector.

### RNA Interference

The target sequences of the FX shRNAs were as follows: sh-FX-1: 5′-GACTGTGACCAGTTCTGCCACGAGGAACA-3′, sh-FX-2: 5′-TTCAAGGACACCTACTTCGTGACAGGCAT-3′. The target sequence of the CASC2c shRNA was 5′-AGACACACACCACACCTCAAATATA-3′. All these DNA segments were synthesized by Sangon Biotech Company and inserted into a pSuper Vector.

### Transient Transfection and Lentivirus Infection

Transient transfection of miRNA mimics and plasmids was performed according to the manufacturer’s manual using lipofectamine 3000 reagent (Thermo Fisher Scientific, L3000015). The lentivirus system purchased from Invitrogen contained four plasmids: pLVX-mCherry-N1, pLP1, pLP2, and pLP/VSVG. FX was constructed in pLVX-mCherry-N1 and transfected into 293FT cells with pLP1, pLP2, and pLP/VSVG. The cellular supernatants were harvested after 48 and 72 h and ultracentrifugation to collect the lentivirus. We infected GL261 cells with lentivirus and screened positive cells with puromycin (Sigma-Aldrich). Then, the cells were cultured in DMEM with 10% FBS (HyClone).

### Real-Time PCR Analysis of miRNA and mRNA

Total RNA was extracted from cultured cells using the TRI reagent (Molecular Research Center, MRC). Total RNA (2 µg) was reverse transcribed to cDNA using the RevertAid First Strand cDNA Synthesis Kit (Thermo Fisher Scientific) according to the manufacturer’s procedure. Real-time PCR was performed using SYBR Green PCR kits (Bimake). miRNA was reverse transcribed to cDNA using a miScript reverse transcription kit (GenePharma). Expression of miRNA was measured by real-time PCR using the miRNA Real-Time PCR Assay Kit (GenePharma). The sequences of the primers are listed in Table S1 in Supplementary Material.

### Western Blot

Western blot analysis was conducted according to the standard procedure. Cells were lysed using ice-cold RIPA buffer containing protease inhibitor cocktail (Bimake) and phosphatase inhibitor (Bimake). Proteins were separated by sodium dodecyl sulfate-polyacrylamide gel electrophoresis and analyzed by immunoblotting. Signals were detected using chemiluminescent HRP substrate (Millipore). The primary antibodies used were as follows: ERK1/2, p-ERK1/2 (Thr202/Tyr204), p-AKT (Ser473) (Cell Signaling Technology), FX, Iba1 (Thermo Fisher Scientific), Flag (Sigma-Aldrich), MYC, and GAPDH (Proteintech).

### Co-Immunoprecipitation

HEK293 cells were lysed with IP lysis buffer (10 mM Tris–HCl pH 7.5; 300 mM NaCl; 10 mM EDTA; 0.5% Triton X-100) supplemented with protease inhibitor cocktail and phosphatase inhibitor. Cell lysates were incubated with Flag antibody for 12 h at 4°C. Then, the solution was incubated with Protein G beads (Thermo Fisher Scientific) for 4 h at 4°C. After the beads were washed and boiled, the supernatants were collected for Western blot detection.

### Monocytes/Macrophages Chemotaxis Assays

THP-1 cells and mouse monocytes/macrophages were cultured in the RPMI-1640 (HyClone) with 10% FBS (HyClone). THP-1 cells were primed with 50 nM phorbol 12-myristate 13-acetate (PMA, Sigma) for 48 h to become monocyte-derived macrophages. Chemotaxis assays assessing cell chemotactic potential were performed in 24-well plates with an 8-µm aperture. A total of 5 × 10^5^ primed THP-1 cells or mouse monocytes/macrophages were cultured in the upper chamber, and culture supernatants from GBM cells were added to the lower chamber and cultured for 24 h. Cells in the lower chamber were fixed with 4% paraformaldehyde and stained with crystal violet. Photos of the cells were captured using a microscope system (Olympus).

### Immunofluorescence Staining and Confocal Laser Scanning

For analysis of the correlation between FX and Iba1, frozen astrocytoma tissue sections were permeated with 0.25% Triton X-100 for 10 min and blocked with 10% goat serum for 30 min. Then, the sections were stained with anti-FX (Thermo Fisher Scientific) and anti-Iba1 (Thermo Fisher Scientific) antibodies for 12 h at 4°C followed by staining with Alexa Fluor 488- and 594-conjugated antibodies (Thermo Fisher Scientific, A11029, A27016). After staining with DAPI (Beyotime Biotechnology, C1002), the sections were mounted by anti-fluorescence quenching agent. Confocal analysis was performed on the Ultra-VIEW VoX system (PerkinElmer) according to the manufacturer’s instructions.

### Immunohistochemistry

Human astrocytoma and mouse orthotopic tumor paraffin sections were dewaxed, rehydrated, and subjected to antigen retrieval. Sections were blocked with 3% hydrogen peroxide for 10 min and normal goat serum for 1 h at room temperature. Then, the sections were incubated with anti-FX (Thermo Fisher Scientific), Iba1 (Thermo Fisher Scientific), CD163, and CD11c (Proteintech) antibodies for 12 h at 4°C and incubated with biotinylated secondary antibody (Maxim Biotechnologies) for 30 min at room temperature followed by streptavidin-conjugated HRP (Maxim Biotechnologies) for 30 min. Staining was visualized with 3,3′-diaminobenzidine (Maxim Biotechnologies) and counterstained with hematoxylin. The immunohistochemical scoring was performed according to the Konno’s criteria (24). The staining index (0–12) of FX was determined by multiplying the score of staining intensity with score of positive area. The staining intensity was scored as 0, negative; 1, weak; 2, moderate; and 3, intense. The positive area was defined as 0, less than 5%; 1, 6–25%; 2, 26–50%; 3, 51–75%; and 4, greater than 75%. FX expression quantification was analyzed by Image Pro Plus vision 6.0, and the IOD was used for drawing. Iba1, CD11c, and CD163 expression were quantified as follows: the number of Iba1^+^ cells, CD11c^+^ cells, and CD163^+^ cells were counted in three random images from a single section and the number per square millimeter was used for drawing.

### CCK8, EDU Incorporation, and Transwell Assay

The CCK8 (Bimake) and EDU incorporation assays (Ribobio) were performed according to the manufacturers’ procedures. Cell viability was assessed by a CCK8 assay as previously described (25). The proliferation ability of astrocytoma cells was detected by an EDU incorporation assay as previously described (26). The invasive ability of astrocytoma cells was tested by a transwell assay (Corning Inc.) as previously described (25).

### Intracranial Implantation Mouse Model

All animal experiments were approved by the Animal Care and Use Committee of Central South University. Five-week-old female C57BL/6 mice were anesthetized with intraperitoneal sodium pentobarbital (40 mg/kg) and fixed in a stereotaxic instrument. Then, an incision was made on the midline of the mouse head, and a hole was drilled in the right hemisphere at AP = +1 mm and ML = −2.5 mm from bregma. Five microliters of 10^6^ cells were injected into the brain at a depth of −3.5 mm from the dura. Mouse weight and survival were recorded daily. After the mice were sacrificed, the whole brains were fixed with 4% paraformaldehyde. HE staining and immunohistochemistry were performed according to the standard procedures.

### Flow Cytometry

THP-1 cells were treated with 50 ng/ml PMA (Sigma) to differentiate into M0 macrophages. Then PMA-primed THP-1 cells were treated with recombinant IL-4 (50 ng/ml, Novoprotein) and IL-13 (20 ng/ml, Novoprotein) to polarization to M2 macrophages, or with LPS (20 ng/ml, Sigma) to M1 macrophages. Cells were harvested at 4°C and blocked nonspecific binding with 5 µl FcR blocker (BioLegend). Cell surfaces were stained with anti-CD11b-FITC, anti-CD206-APC, and anti-CD80-PE (BioLegend) for 20 min on ice, and fluorescence was measured by FACSCanto II (BD Biosciences). CD11b^+^CD206^+^ cells represented as M2 macrophages and CD11b^+^CD80^+^ cells represented as M1 macrophages. Data were analyzed with Flow Jo software.

### Statistical Analysis

All the experiments were repeated at least three times, and the representative data are shown. The statistical analysis was performed using GraphPad Prism 5 and SPSS version 17.0. Data analysis was performed with Student’s *t*-test and one-way ANOVA and presented as the mean ± SEM. *p* Values less than 0.05 were considered significant.

## Results

### FX Promoted the Growth of GBM Cells *In Vivo* but Did Not Affect Cell Proliferation *In Vitro*

**Figure 1:**
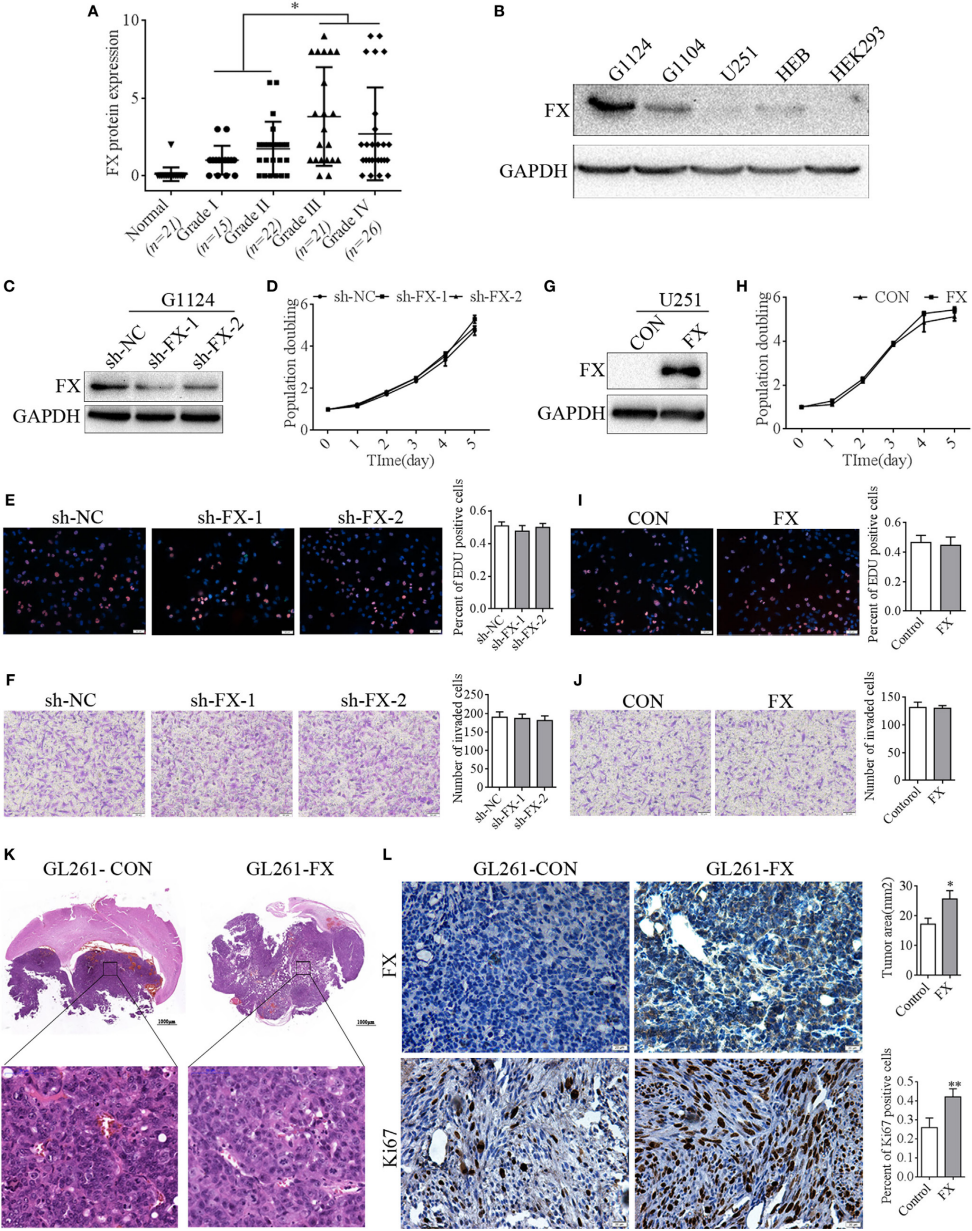
Factor X (FX) promoted the growth of glioblastoma multiforme (GBM) cells *in vivo* but not *in vitro*. **(A)** Immunohistochemistry detected the FX expression in astrocytoma of different WHO grades. Normal brains (*n* = 21); grade I (*n* = 15); grade II (*n* = 22); grade III (*n* = 21); and grade IV (*n* = 26). Data are presented as the mean ± SEM (*p < 0.05). **(B)** Western blotting detected FX expression in primary cultured GBM cells (G1124, G1104) and other cell lines (HEB, U251, and HEK293). **(C)** Western blotting showed that knockdown of FX decreased FX expression in G1124 cells. **(D–F)** Knockdown of FX did not affect G1124 cell viability, proliferation, and invasion measured by CCK8 **(D)**, EDU incorporation assays **(E)**, and transwell assays **(F)**. Data are presented as the mean ± SEM (*p < 0.05). **(G)** Western blotting showed that overexpression of FX increased FX expression in U251 cells. **(H–J)** FX overexpression did not affect U251 cell viability, proliferation, and invasion measured by CCK8 **(H)**, EDU incorporation assays **(I)**, and transwell assays **(J)**. Data are presented as mean ± SEM (*p < 0.05). **(K)** GL261 cells that were infected with lentivirus (GL261-CON and GL261-FX) were intracranially injected into the corpus striatum of C57BL/6 mice. HE staining showed that the mice transplanted with GL261-FX cells exhibited larger tumor volumes. Data are presented as the mean ± SEM (*p < 0.05). **(L)** Immunohistochemistry detected FX and Ki67 expression in intracranial xenografts.

Our previous research reported that *FX* was a hypomethylation gene highly expressed in glioma (21). In this study, to ascertain the potential role of FX in the pathogenesis of astrocytoma, astrocytoma tissue sections of different WHO grades were detected by immunohistochemistry with an FX antibody. FX was not detected in human normal brain tissues, while FX expression was increased significantly in astrocytoma tissues and dramatically elevated in high-grade astrocytoma (WHO III and IV grade) compared with that in low grade (WHO I and II grade) astrocytoma (Figure 1A). To confirm the expression of FX in cell lines, FX protein levels were measured in primary cultured GBM cells (G1124, G1104), U251, normal human glial cells (HEB), and HEK293 cells. FX was highly expressed in G1124 cells and, to a lesser extent, in G1104 cells, but it was nearly undetectable in U251, HEB, and HEK293 cells (Figure 1B). Unlike GFP alone, which filled whole cells, FX-GFP localized primarily to the trans-Golgi network and vesicles (Figure S1A in Supplementary Material), suggesting that FX was a secreted protein. To examine the role of FX in GBM cells, FX-shRNAs were transfected into G1124 cells, and decreased FX expression was detected by Western blot (Figure 1C). Knockdown of FX did not affect the proliferation of G1124 cells measured using CCK8 (Figure 1D) and EDU assays (Figure 1E). Matrigel invasion assays showed that the invasion of G1124 cells was not influenced by FX (Figure 1F). Furthermore, overexpressing FX in U251 cells did not influence the cell proliferation and invasion (Figures 1G–J). Next, GL261 cells that stably overexpressed FX by lentiviral infection were constructed and transplanted into the corpus striatum zone of C57BL/6 mice to form intracranial orthotopic GL261 xenografts. The expression of FX was higher in GL261-FX cells than in GL261-CON cells (Figure S1B in Supplementary Material). These two cell lines had the same proliferation rate despite FX expression (Figure S1C in Supplementary Material). By contrast, overexpression of FX significantly promoted the growth of intracranial orthotopic GL261 xenografts measured by HE staining (Figure 1K). Overexpression of FX significantly enhanced the expression of Ki-67 as shown by immunohistochemistry (Figure 1L), which suggested that FX promoted cell proliferation *in vivo*. These results indicated that increasing FX expression accelerated glioma tumor growth *in vivo*, while FX did not affect the proliferation and invasion *in vitro*.

### High Expression of FX Was Positively Correlated With TAM Density in GBM

**Figure 2:**
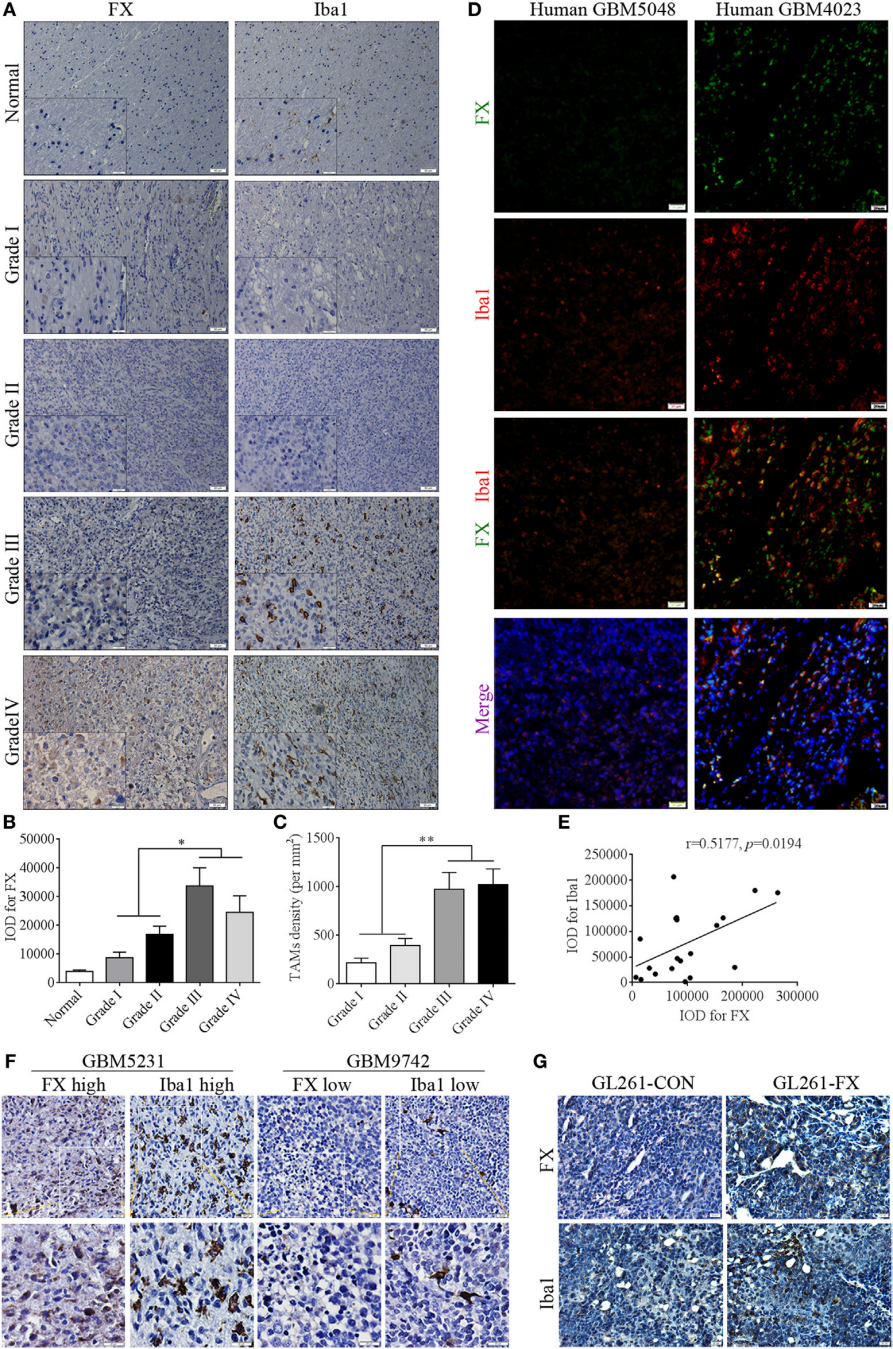
Factor X (FX) expression was positively correlated with tumor-associated macrophage (TAM) density in human glioblastoma multiformes (GBMs). **(A)** Immunohistochemistry showed the positive correlation between FX and the TAM marker Iba1 (red) in human normal brains (*n* = 21) and astrocytoma of different WHO grades. Grade I (*n* = 15); grade II (*n* = 22); grade III (*n* = 21); and grade IV (*n* = 26). **(B)** The representative histogram of average IOD of FX in panel **(A)**. **(C)** The representative histogram of average number of TAMs per square millimeter in panel **(A)**. **(D)** Immunofluorescence analysis showed the positive correlation between FX (green) and Iba1 (red) expression in human GBM tissues (*n* = 20). **(E)** The correlation analysis of IOD of FX and Iba1 in panel **(D)**. **(F)** Immunohistochemical staining showed that higher FX expression was accompanied by more TAM infiltration in consecutive paraffin sections of human GBM tissues (*n* = 26). **(G)** Immunohistochemical staining showed there were more TAMs in GL261-FX-derived xenografted tumor than in GL261-CON tumor.

Recent studies indicated that FX is a secretion protein that plays a key role in blood coagulation by converting prothrombin to thrombin, leading to the formation of a fibrin clot. In addition, integrin αMβ2 is a key receptor of FX in mediating cell migration (27). Integrin subunit alpha M, also known as cluster of differentiation molecule 11b (CD11b), encodes the integrin alpha M chain, which is frequently used as a marker of macrophages. Therefore, we speculated that FX may influence the chemotaxis of macrophages to facilitate tumor growth in the brain. To address the correlation between FX and TAMs in human astrocytoma, astrocytoma sections were immunostained with FX and the TAM marker Iba1. In WHO grades I and II astrocytoma, TAM and FX expression was scarce, while in WHO grades III and IV astrocytoma, TAM and FX were highly expressed (Figures 2A–C). Immunofluorescence and immunohistochemistry assays further indicated that higher FX expression was accompanied by more TAM infiltration, while lower FX expression in human GBM tissues had less Iba1 staining (Figures 2D–F). In GL261-FX-derived xenografted tumors, TAMs were more abundant than that in GL261-CON-derived tumors (Figure 2G). These results suggested a positive correlation between FX protein levels and TAM density in GBMs, and FX may contribute to the infiltration of TAMs to promote tumor growth *in vivo*.

### FX Exhibited a Potent Capacity to Recruit Macrophages/Monocytes

**Figure 3:**
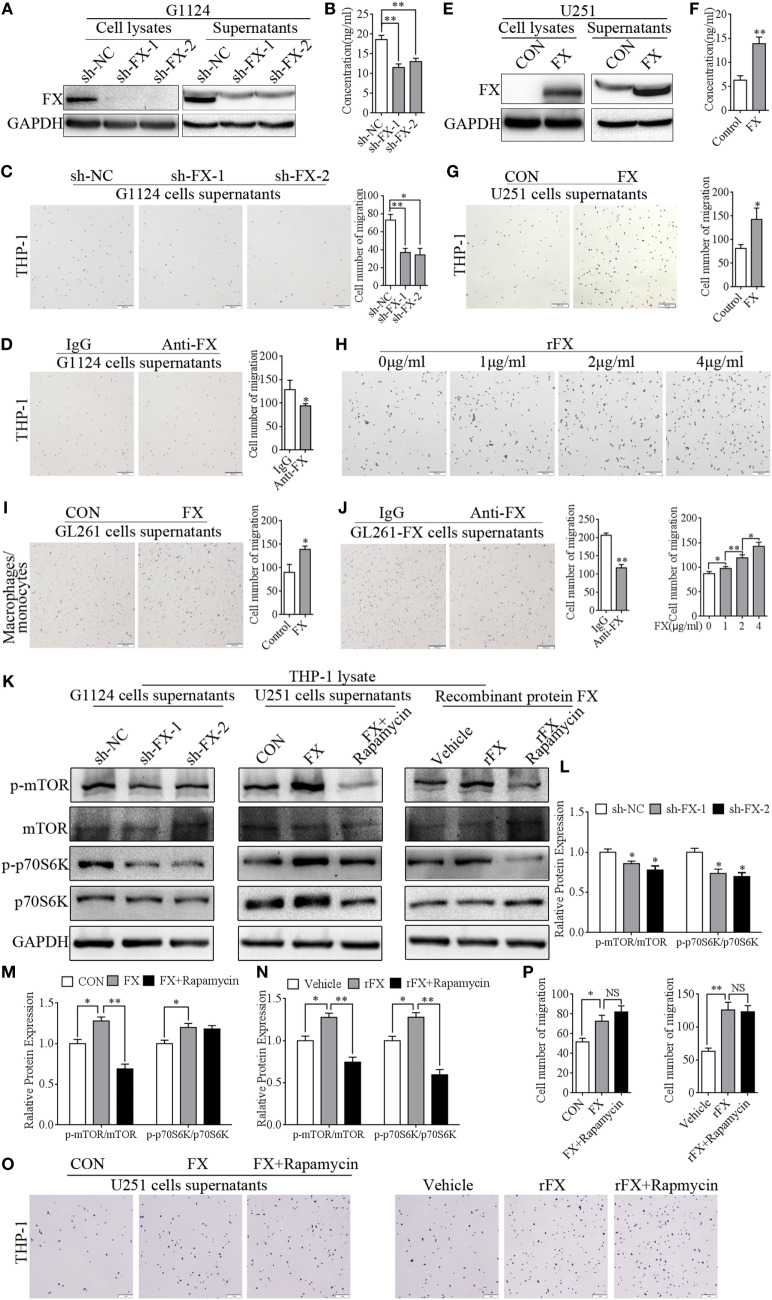
Factor X (FX) had a chemotactic ability to recruit macrophages/monocytes. **(A)** Western blotting showed that FX expression decreased in G1124 cell lysates and supernatants after knockdown of FX (mean ± SEM, **p < 0.01). **(B)** ELISA demonstrated that the secretion of FX was reduced in G1124 cell supernatants after knockdown of FX (mean ± SEM, **p < 0.01). **(C)** Representative images of macrophage-like THP-1 cells that migrated toward the supernatants from G1124 cells transfected with sh-NC and sh-FX in transwell assays. After knockdown of FX in G1124 cells, the number of migrating cells decreased (mean ± SEM, *p < 0.05, **p < 0.01). **(D)** Representative images of THP-1 cells that migrated toward G1124 cell media preincubated with anti-FX (10 µg/ml) antibody or IgG. After preincubation with anti-FX antibody, the number of migrating cells decreased (mean ± SEM, *p < 0.05). **(E)** Western blotting detected that FX expression increased in U251 cell lysates and supernatants after transfection with pcDNA3.1-FX. **(F)** ELISA analysis showed FX secretion in U251 cell supernatants increased after transfection with pcDNA3.1-FX (mean ± SEM, **p < 0.01). **(G)** The number of THP-1 cells migrating toward media from FX-overexpressing U251 cells was greater than that in the control by transwell assays (mean ± SEM, *p < 0.05). **(H)** THP-1 cells migrating toward different concentrations of recombinant FX (rFX) by transwell assays showed that FX attracted THP-1 cells in a dose-dependent manner (mean ± SEM, *p < 0.05, **p < 0.01). **(I)** Representative images of mouse macrophages/monocytes that migrated toward supernatants from GL261 transfected with FX-overexpressing or control vector. The results showed that the number of macrophages/monocytes migrated toward GL261 cell supernatants were increased when FX was overexpressed (mean ± SEM, *p < 0.05). **(J)** The number of recruitment mouse macrophages/monocytes was decreased when GL261-FX cell supernatants were preincubated with anti-FX (10 µg/ml) antibody (mean ± SEM, **p < 0.01). **(K)** p-mTOR, mTOR, p-p70S6K, and p70S6K in THP-1 cells were detected by western blot when phorbol 12-myristate 13-acetate (PMA)-primed THP-1 cells were treated with supernatants with or without rapamycin. **(L)** Representative histogram of analysis of p-mTOR/mTOR and p-p70S6K/p70S6K when THP-1 treated with G1124 cell supernatants knockdown of FX in panel **(K)**. **(M)** Representative histogram of analysis of p-mTOR/mTOR and p-p70S6K/p70S6K when THP-1 treated with U251 cell supernatants overexpression of FX in panel **(K)**. **(N)** Representative histogram of analysis of p-mTOR/mTOR and p-p70S6K/p70S6K when THP-1 treated with recombinant protein FX in panel **(K)**. **(O)** THP-1 cells migrating toward cells supernatants with or without rapamycin. **(P)** The histogram of cell number of migrated cells in panel **(O)**.

To examine whether FX function as a potent chemoattractant of TAMs, a series of migration assays were used to examine the capacity of FX to attract macrophages. Knockdown of FX in primary GBM G1124 cells by two specific shRNAs significantly decreased FX expression in the cells and culture supernatants as measured by Western blot and ELISA (Figures 3A,B). The culture supernatants from G1124 cells which knockdown of FX reduced the migration of PMA-primed macrophage-like THP-1 cells (Figure 3C). Preincubation of G1124 cell culture supernatants with an anti-FX antibody attenuated the chemotaxis effect of FX on macrophage migration (Figure 3D). By contrast, overexpression of FX in U251 cells increased FX expression in the cells and culture supernatants (Figures 3E,F), and the culture supernatants markedly enhanced the ability to recruit PMA-primed macrophage-like THP-1 cells (Figure 3G). To further explore whether the FX-mediated chemotactic effect on macrophage migration was dose dependent, we performed the chemotaxis experiments with different concentrations of recombinant FX (rFX) protein. The migration of macrophages toward FX was significantly enhanced as the rFX protein increased (Figure 3H), indicating that FX attracted macrophages in a dose-dependent manner. The capacity of FX to recruit macrophages was further demonstrated by the isolated mouse primary macrophages/monocytes in migration and invasion assays. Overexpressing FX in GL261 cells recruited more macrophages/monocytes to the lower chamber of the transwells (Figure 3I). The chemotactic ability of FX was attenuated by FX antibody (Figure 3J). The above data demonstrated that FX secreted by GBM cells exhibited a potent capacity to recruit macrophages/monocytes. To address whether FX recruited macrophages in an mTOR-dependent or -independent manner, we treated PMA-primed macrophage-like THP-1 cells with the culture supernatants from G1124 cells in which FX had been knocked down or culture supernatants from U251 cells in which FX was overexpressed and detected the expression of p-mTOR and p-p70S6K of THP-1 cells. When PMA-primed THP-1 cells were treated with G1124-sh-FX cells supernatants, p-mTOR and p-p70S6K decreased. By contrast, when PMA-primed THP-1 cells were incubated with U251-FX cells supernatants, p-mTOR and p-P70S6K increased. Moreover, when PMA-primed THP-1 cells were treated with recombinant protein FX, p-mTOR and p-p70S6K increased (Figures 3K–N). In addition, inhibitor of mTOR rapamycin suppressed p-mTOR and p-p70S6K in THP-1 cells. But rapamycin did not inhibit the chemotaxis of FX on PMA-primed THP-1 cells (Figures 3O,P) suggested that FX recruited macrophages in an mTOR-independent manner.

### FX Overexpression in GBM Cells Specifically Increased M2 Tumor-Supportive TAMs

**Figure 4:**
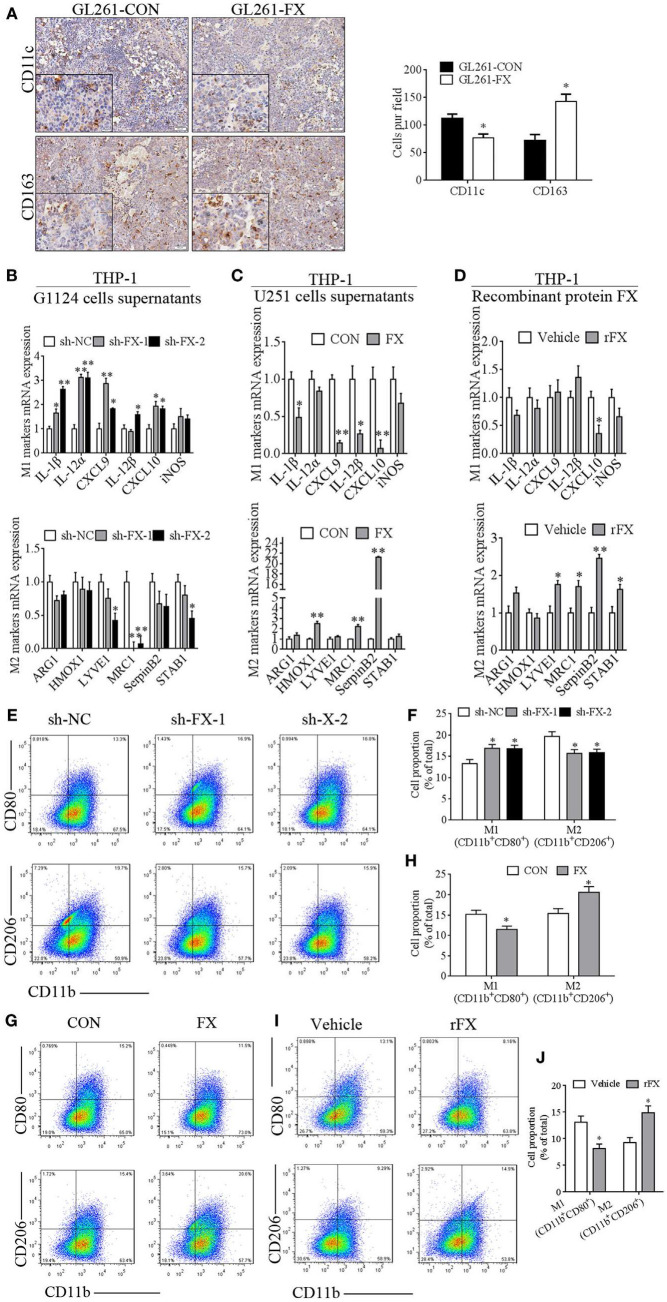
Factor X (FX) promoted macrophage polarization to the M2 subtype. **(A)** Immunohistochemical staining of GL261-CON- and GL261-FX-derived xenografted tumors by CD11c and CD163 antibody. In GL261-FX-derived xenografted tumors, there was more CD163 staining and less CD11c staining than in the GL261-CON tumor. **(B)** Macrophage subtype markers were measured by real-time PCR after treatment with supernatants from G1124 cells transfected with sh-NC or sh-FX. In the sh-FX group, IL-1β, IL-12α, CXCL9, IL-12β, and CXCL10 levels were higher, while LYVE1, MRC1, and STAB1 levels were lower compared those in the sh-NC group (mean ± SEM, *p < 0.05, **p < 0.01). **(C)** Supernatants from U251 cells transfected with FX or control vectors were added to THP-1 cells, and the markers’ mRNAs were detected by real-time PCR. IL-1β, CXCL9, IL-12β, and CXCL10 levels were decreased, while HMOX1, MRC1, and SerpinB2 levels were increased when FX was overexpressed (mean ± SEM, *p < 0.05, **p < 0.01). **(D)** THP-1 cells were treated with recombinant protein FX and markers’ mRNAs were measured by real-time PCR. CXCL10 expression decreased, while M2 markers (LYVE1, MRC1, SerpinB2, and STAB1) increased after treatment with recombinant FX (rFX) (mean ± SEM, *p < 0.05, **p < 0.01). **(E–J)** CD80 and CD206 of phorbol 12-myristate 13-acetate (PMA)-primed THP-1 cells were detected by flow cytometry. **(E)** The proportion of M1 macrophages increased and M2 macrophages decreased when THP-1 cells were treated with G1124-sh-FX cell supernatants. **(F)** Bar graph of CD11b^+^CD80^+^ and CD11b^+^CD206^+^ cell proportion in panel **(E)**. **(G)** The proportion of M1 macrophages decreased and M2 macrophages increased when THP-1 cells were treated with U251-FX cell supernatants compared with U251-CON. **(H)** Bar graph of CD11b^+^CD80^+^ and CD11b^+^CD206^+^ cell proportion in panel **(G)**. **(I)** The proportion of M1 macrophages decreased and M2 macrophages increased when THP-1 cells were treated with FX recombinant protein. **(J)** Bar graph of CD11b^+^CD80^+^ and CD11b^+^CD206^+^ cell proportion in **(I)**.

To address which subtype of TAMs was recruited or maintained by FX in GBM, we applied M1-specific marker (CD11c) and M2-specific marker (CD163) to distinguish TAMs in intracranial orthotopic GL261-CON- and GL261-FX-derived xenografts. There was more CD163 staining and less CD11c staining in the GL261-FX-derived tumors than that in the Gl261-CON tumors, which indicated that FX increased M2 subtype macrophages (Figure 4A). In addition, we treated PMA-primed macrophage-like THP-1 cells with the culture supernatants from G1124 cells in which FX had been knocked down or culture supernatants from U251 cells in which FX was overexpressed and detected the expression of M1 markers (IL-1β, IL-12α, CXCL9, IL-12β, CXCL10, and iNOS) and M2 markers (ARG1, HMOX1, LYVE1, MRC1, SerpinB2, and STAB1). When THP-1 cells were treated with G1124-sh-FX cell supernatants, IL-1β, IL-12α, CXCL9, IL-12β, and CXCL10 levels were increased, while LYVE1, MRC1, and STAB1 levels were decreased (Figure 4B). By contrast, when THP-1 cells were incubated with U251-FX cell supernatants, IL-1β, CXCL9, IL-12β, and CXCL10 levels were decreased, while HMOX1, MRC1, and SerpinB2 levels were increased (Figure 4C). When THP-1 cells were treated with recombinant protein FX (4 µg/ml), the M1 marker CXCL10 expression was also decreased, while M2 markers (LYVE1, MRC1, SerpinB2, and STAB1) were increased (Figure 4D). Flow cytometry using CD80 as M1 marker and CD206 as M2 marker also demonstrated that when THP-1 cells were treated with G1124-sh-FX cell supernatants, the proportion of M1 macrophages increased and M2 macrophages decreased (Figures 4E,F). By contrast, when THP-1 cells were treated with U251-FX cell supernatants or recombinant protein FX, the proportion of M1 macrophages decreased and M2 macrophages increased (Figures 4G–J). Collectively, these data indicated that FX mainly promoted macrophages polarization to the M2 subtype.

### FX Recruited and Influenced the Polarization of Macrophages Through ERK1/2 and AKT

**Figure 5:**
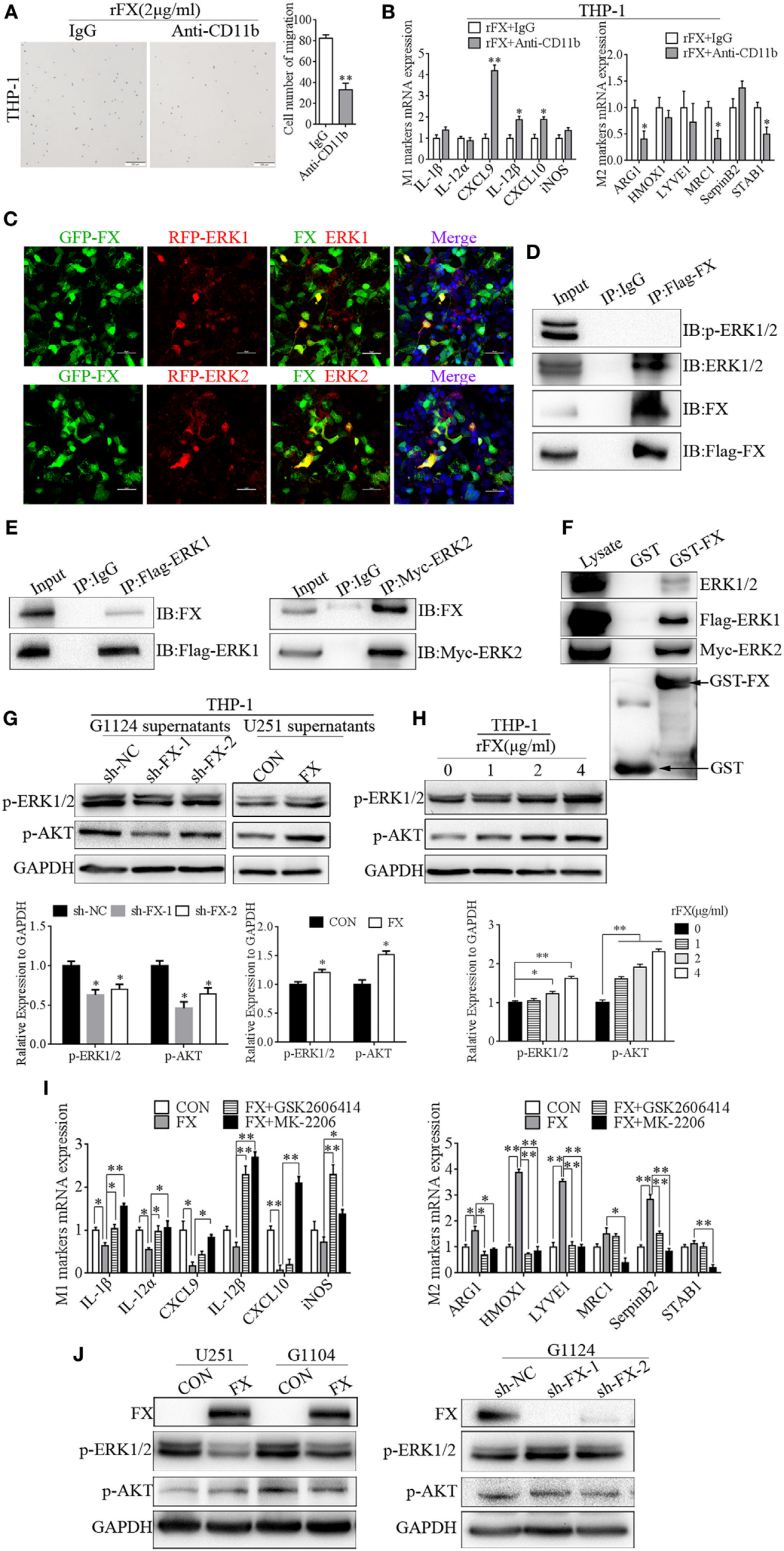
Factor X (FX) recruited and influenced macrophage polarization through extracellular signal-related kinase (ERK)1/2 and AKT. **(A)** Transwell assays showed that THP-1 cells’ migration toward FX decreased after preincubation with a CD11b antibody (mean ± SEM, **p < 0.01). **(B)** Real-time PCR analysis of M1 and M2 markers in THP-1 cells after the cells were treated with CD11b antibody (mean ± SEM, *p < 0.05, **p < 0.01). **(C)** Confocal fluorescence microscopy of HEK293 cells cotransfected with GFP-FX (green) and RFP-ERK1 (red) or RFP-ERK2 (red) showed the co-localization of FX with ERK1 and ERK2. **(D)** Co-immunoprecipitation showed the interaction between FX and endogenous ERK1/2 in HEK293 cells after transfection with Flag-FX. **(E)** Co-immunoprecipitation showed the interaction between exogenous FX and ERK1/2 in HEK293 cells after cotransfection with pcDNA3.1-FX and flag-ERK1 or myc-ERK2. **(F)** GST pull-down assays showed the binding of FX and ERK1 or ERK2. **(G)** p-ERK1/2 and p-AKT were decreased when THP-1 cells were treated with G1124 cell culture supernatants after knockdown of FX; p-ERK1/2 and p-AKT were increased when THP-1 cells were treated with U251 cell culture supernatants after overexpression of FX. **(H)** p-ERK1/2 and p-AKT increased in a concentration-dependent manner with the stimulation of recombinant FX (rFX). **(I)** THP-1 cells incubated with G1124-FX cell supernatants supplemented with or without p-ERK1/2 (GSK2606414) or p-AKT (MK2206) inhibitors. M1 macrophage marker levels increased, while M2 macrophage marker levels decreased with these two inhibitors (mean ± SEM, *p < 0.05, **p < 0.01). **(J)** p-ERK1/2 and p-AKT were detected by Western blotting in U251 and G1104 cell overexpression of FX and G1124 cell knockdown of FX.

To further investigate the molecular mechanism underlying the FX-mediated recruitment and polarization of TAMs, we used CD11b antibody to block the integrin signaling on PMA-primed macrophage-like THP-1 cells and found that CD11b antibody significantly reduced the chemotaxis and mobility of the THP-1 cells induced by rFX protein (Figure 5A). At the same time, CD11b antibody increased the expression of the M1 markers (CXCL9, IL-12β, and CXCL10) and decreased the expression of M2 markers (ARG1, MRC1, and STAB1) (Figure 5B).

Furthermore, ERK1/2 and AKT played important roles in the regulation of M2 macrophage cell-specific genes such as ARG-1 and MRC-1 (28). Prediction with bioinformatics software Scansite 3.0 found that FX may interact with ERK1/2 (Figure S2A in Supplementary Material). Therefore, we detected whether FX colocalized with ERK1/2. GFP-FX and RFP-ERK1 or RFP-ERK2 were constructed and cotransfected into HEK293 cells. As shown in Figure 5C, confocal fluorescence microscopy displayed that FX was colocalized with ERK1 and ERK2. To determine whether FX and ERK1/2 could be co-immunoprecipitated from cells, we constructed a full-length Flag-FX vector and transfected in HEK293 cells. FX was co-immunoprecipitated with endogenous ERK1 and ERK2 but not p-ERK1 and p-ERK2 (Figure 5D). In addition, FX was co-immunoprecipitated with exogenous ERK1 and ERK2 (Figure 5E). GST with an FX fusion protein was constructed, and a GST pull-down assay demonstrated that FX bound to ERK1 and ERK2 (Figure 5F). We also used co-immunoprecipitation to detect whether AKT interacted with FX, and the results suggested that AKT did not interact with FX (Figure S2B in Supplementary Material).

We further investigated whether FX mediate macrophage polarization through ERK1/2 and AKT. p-ERK and p-AKT were decreased when THP-1 cells were treated with cell culture supernatants from G1124 cells in which FX was knocked down, while when THP-1 cells were treated with cell culture supernatants from U251 cells which FX was overexpressed, p-ERK1/2 and p-AKT were increased (Figure 5G). p-ERK1/2 and p-AKT also increased when THP-1 cells were treated with rFX protein in a concentration-dependent manner (Figure 5H). Next, we used an inhibitor of p-ERK1/2 (GSK2606414) or p-AKT (MK2206) to inhibit p-ERK1/2 or p-AKT, respectively, in THP-1 cells and measured the markers of M1 and M2 macrophages. When THP-1 cell were incubated with FX-overexpression-U251 cell supernatants supplemented with these two inhibitors, both inhibitors reversed the M1 and M2 macrophage marker expression induced by FX (Figure 5I). Subsequently, we also examined the effect of FX on intracellular p-ERK1/2 and p-AKT in GBM cells. Overexpression of FX decreased p-ERK1/2 in G1104 and U251 cells, while knockdown of FX increased p-ERK1/2 in G1124 cells but had almost no influence on p-AKT (Figure 5J). These results demonstrated that FX bound to ERK1 and ERK2 and is involved in regulating ERK1/2 phosphorylation.

Taken together, these findings indicate that FX bound to ERK1/2 and inhibited p-ERK1/2 expression in GBM cells. Moreover, FX was secreted from GBM cells and bond to integrin αMβ2 on the surfaces of macrophages to recruit them to the tumors. FX raised expression of p-ERK1/2 and p-AKT in macrophages to induce them to M2-type polarization.

### miR-338-3p Targeted FX to Suppress Macrophage Migration

**Figure 6:**
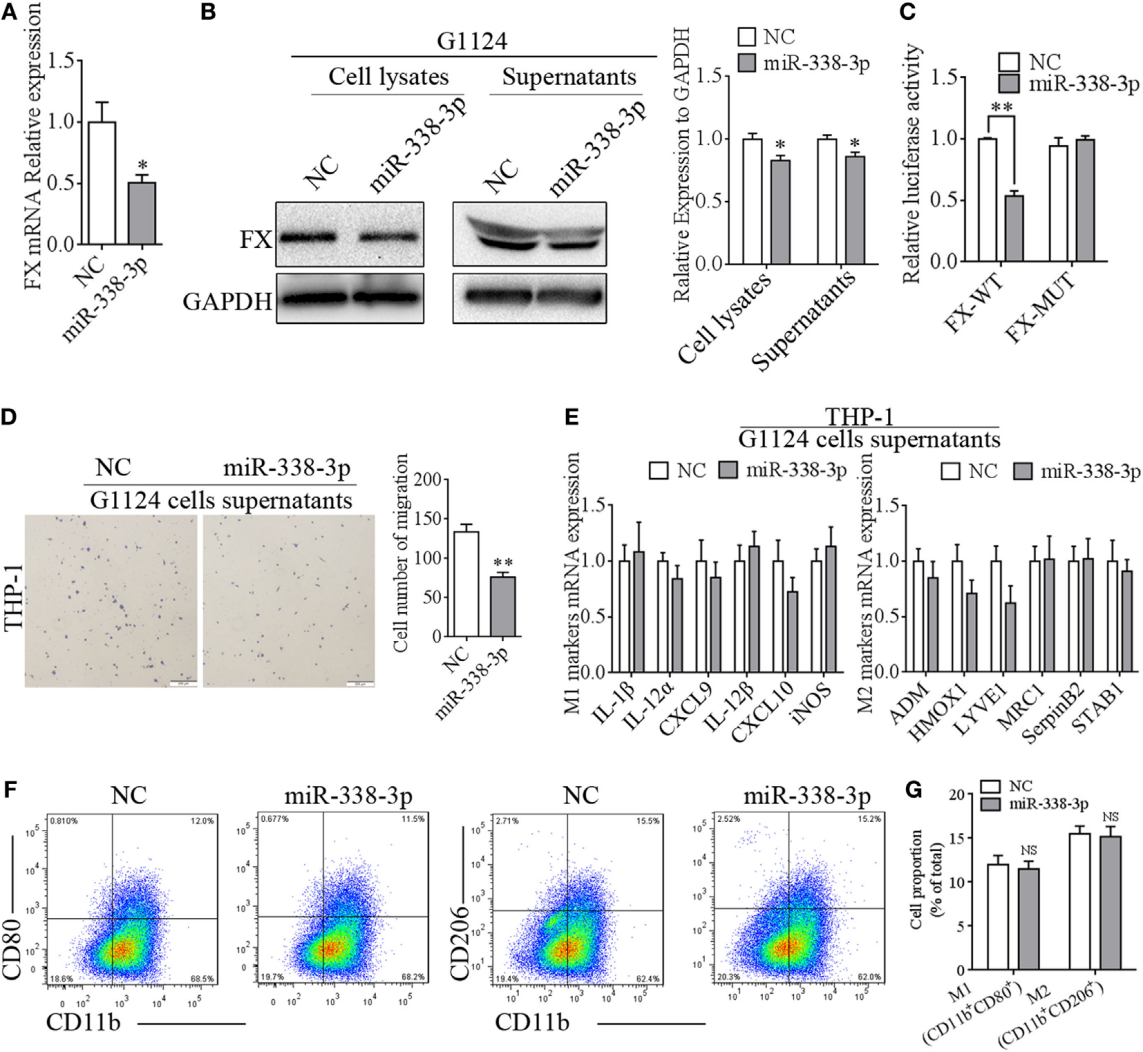
miR-338-3p targeted factor X (FX) to suppress macrophage migration. **(A)** Real-time PCR detected that the mRNA expression of FX decreased after transfection with miR-338-3p mimics in G1124 cells (mean ± SEM, *p < 0.05). **(B)** Western blotting detected reduced FX expression in cell lysates and supernatants after G1124 cells were transfected with miR-338-3p mimics. **(C)** The luciferase reporter assay showed that miR-338-3p decreased luciferase activity (mean ± SEM, **p < 0.01). **(D)** The number of phorbol 12-myristate 13-acetate (PMA)-primed THP-1 cells migrating toward G1124 cell supernatants decreased when G1124 cells were transfected with miR-338-3p mimics (mean ± SEM, *p < 0.01). **(E)** Real-time PCR detected the expression of M1 and M2 markers in THP-1 cells after incubation with G1124 cell supernatants transfected with NC or miR-338-3p mimics (mean ± SEM). **(F,G)** Flow cytometry analysis showed that the proportion of CD11b^+^CD80^+^ M1 macrophages and CD11b^+^CD206^+^ M2 macrophages did not changed in THP-1 cells treated with supernatants from G1124 cells transfected with miR-338-3p compared with NC. **(G)** Bar graph of CD11b^+^CD80^+^ and CD11b^+^CD206^+^ cell proportion in panel **(F)**.

We confirmed that FX recruited macrophages to the tumors and influenced polarization of macrophages through p-ERK1/2 and p-AKT. Then, we turned our attention to the molecule that affected the expression of FX in GBM cells. By prediction with TargetScan,^1^ FX may be targeted by miR-338-3p (Figure S3A in Supplementary Material); moreover, according to the miRcode,^2^ miR-338-3p was likely to interact with lncRNA CASC2c, which is an onco-RNA acting in tumorigenesis of astrocytoma (Figure S3B in Supplementary Material). miR-338-3p mimics were transfected into G1124 cells that highly expressed FX, and the expression of FX was decreased for mRNA (Figure 6A) and protein (Figure 6B). To ascertain whether FX was the direct target gene of miR-338-3p, FX putative miR338-3p recognition sequences (pmirGLO-FX-WT) and mutant derivatives lacking the binding sequences (pmirGLO-FX-MUT) were cloned downstream of the luciferase gene into the pmirGLO vector and were transfected into HEK293 cells with NC or miR-338-3p mimics (Figure S3C in Supplementary Material). miR-338-3p reduced the luciferase activity when transfected with pmirGLO-FX-WT, while it almost did not influence luciferase activity when transfected with pmirGLO-FX-MUT (Figure 6C). To further validate whether miR-338-3p could regulate the migration of macrophages, the supernatants from G1124 cells transfected with miR-338-3p or NC were harvested to chemoattract THP-1-derived macrophage-like cells. miR-338-3p decreased the number of THP-1 cells that migrated (Figure 6D). However, the M1 markers (IL-1β, IL12A, CXCL9, CXCL10, and iNOS) and M2 markers (ADM, HMOX1, MRC1, SerpinB2, and STAB1) were almost unchanged in THP-1 cells, whether the cells were treated with supernatants from G1124 cells transfected with miR-338-3p or NC (Figure 6E). Flow cytometry also demonstrated that the proportion of CD11b^+^Cd80^+^ M1 macrophages and CD11b^+^CD206^+^ M2 macrophages did not changed in THP-1 cells treated with supernatants from G1124 cells transfected with miR-338-3p compared with NC (Figures 6F,G). These results suggested that miR-338-3p targeted FX and suppressed macrophage migration through FX, but did not affect macrophage polarization.

### CASC2c Regulated FX Expression and Inhibited Macrophage Migration and Polarization

**Figure 7:**
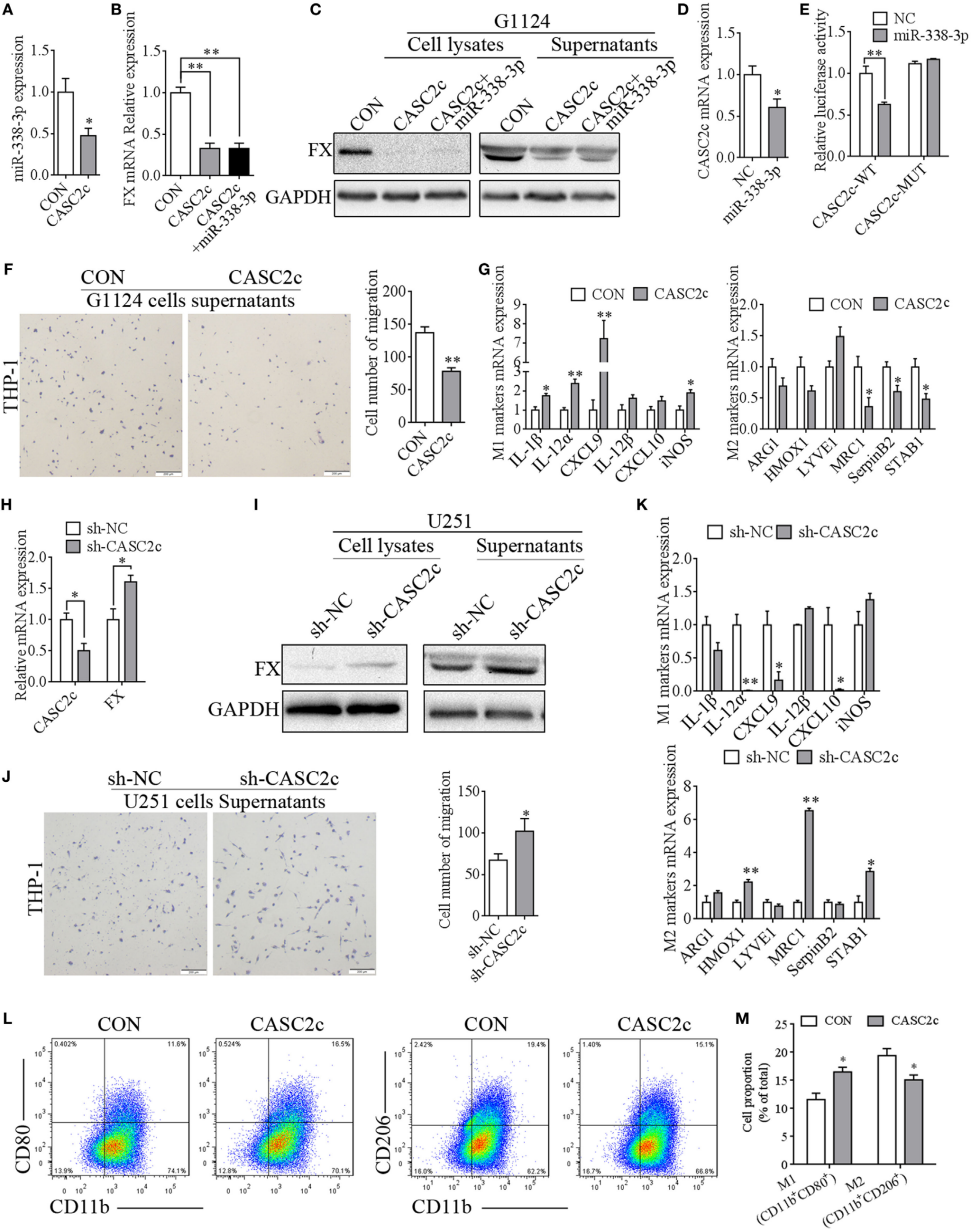
CASC2c regulated factor X (FX) expression and macrophage migration and polarization. **(A)** Real-time PCR detected decreased miR-338-3p expression in G1124 cells when transfected with CASC2c (mean ± SEM, *p < 0.05). **(B)** Real-time PCR detected reduced FX expression in G1124 cells with overexpression of CASC2c (mean ± SEM, **p < 0.01). **(C)** Western blotting detected reduced FX expression in G1124 cells with overexpression of CASC2c. **(D)** Real-time PCR detected decreased CASC2c expression after transfection with miR338-3p mimics (mean ± SEM, *p < 0.05). **(E)** The luciferase reporter assay showed that the effect of miR-338-3p on CASC2c activity (mean ± SEM, **p < 0.01). **(F)** Overexpression of CASC2c in G1124 cells suppressed the migration of THP-1 cells toward G1124 cell supernatants (mean ± SEM, **p < 0.01). **(G)** Real-time PCR detected the M1 markers and M2 markers in THP-1 cells after incubation with G1124 cell supernatants that were transfected with CASC2c or control vector. M2 markers (MRC1, SerpinB2, and STAB1) were decreased, while M1 markers (IL-1β, IL-12α, CXCL9, and iNOS) in THP-1 cells were increased when CASC2c was overexpressed in G1124 cells (mean ± SEM, *p < 0.05, **p < 0.01). **(H)** Knockdown of CASC2c increased FX mRNA expression in U251 cells (mean ± SEM, *p < 0.05). **(I)** Knockdown of CASC2c increased FX protein detected by Western blotting (mean ± SEM, *p < 0.05). **(J)** Knockdown of CASC2c recruited more THP-1 cells measured by transwell assay (mean ± SEM, *p < 0.05). **(K)** M1 markers and M2 markers in THP-1 cells were measured by real-time PCR after incubated with U251 cell supernatants transfected with sh-CASC2c or sh-NC vector. The mRNA expression of M1 markers (IL-12α, CXCL9, and CXCL10) were decreased, while M2 markers (HMOX1, MRC1, and STAB1) were increased when THP-1 cells were incubated with U251 cell supernatants transfected with sh-CASC2c (mean ± SEM, *p < 0.05, **p < 0.01). **(L,M)** Flow cytometry revealed that the proportion of CD11b^+^CD80^+^ M1 macrophages increased and CD11b^+^CD206^+^ M2 macrophages decreased when THP-1 treated with supernatants from G1124 cells transfected with CASC2c compared with control vector. **(M)** Bar graph of CD11b^+^CD80^+^ and CD11b^+^CD206^+^ cell proportion in panel **(L)**.

To confirm whether CASC2c regulate FX by competing with miR-338-3p, CASC2c was transfected into G1124 cells. miR-338-3p expression was decreased (Figure 7A), and FX mRNA and protein expression were also decreased. Moreover, cotransfection of CASC2c and miR-338-3p repressed FX mRNA and protein expression (Figures 7B,C). In turn, miR-338-3p also decreased CASC2c expression (Figure 7D). To verify the direct binding between CASC2c and miR-338-3p, we cloned the recognition sequence of CASC2c (pmirGLO-CASC2c-WT) and a mutated sequence (pmirGLO-CASC2c-MUT) into the pmirGLO vector and transfected HEK293 cells with NC or miR-338-3p mimics (Figure S3D in Supplementary Material). miR-338-3p reduced the luciferase activity when transfected with pmirGLO-CASC2c-WT vector, while miR-338-3p had no effect on luciferase activity when transfected with the pmirGLO-CASC2c-MUT vector (Figure 7E). The above results indicated that CASC2c interacted with miR-338-3p and act as a direct target gene of miR-338-3p. CASC2c potently inhibited FX expression despite the presence of miR-338-3p.

To explore CASC2c’s function on macrophage migration and polarization, the supernatants from G1124 cells transfected with CASC2c were collected to treat PMA-primed THP-1 cells. CASC2c suppressed the migration of macrophages (Figure 7F). M2 markers decreased while M1 markers increased compared with those of control cells (Figure 7G). Conversely, we used sh-CASC2c to knockdown CASC2c mRNA expression to further verify these results. Knockdown of CASC2c increased the mRNA (Figure 7H) and protein (Figure 7I) expression of FX in U251 cells. The number of THP-1 cells that migrated was increased when THP-1 cells were incubated with supernatant from U251 cells with CASC2c knockdown (Figure 7J). The mRNA expression of M1 markers decreased while that of M2 markers increased (Figure 7K). Flow cytometry also showed that the proportion of CD11b^+^Cd80^+^ M1 macrophages increased and CD11b^+^CD206^+^ M2 macrophages decreased when THP-1 treated with supernatants from G1124 cells transfected with CASC2c compared with control vector (Figures 7L,M). These results suggested that CASC2c regulated FX expression and inhibited macrophage migration and polarization to the M2 subtype.

## Discussion

Glioblastoma multiforme often contains a large number of TAMs that constitute a major component of the inflammatory cells in the tumor microenvironment (29). These cells have been implicated in glioma angiogenesis, invasion, local tumor recurrence, and immunosuppression (30). Tumor cells can secrete several chemokines, such as CC chemokine ligand 2, soluble colony-stimulating factor 1, stromal cell-derived factor, hepatocyte growth factor, and periostin (POSTN), to recruit TAMs in cancers (31–34). FXa is activated by the cleavage of the activation peptide by factor IXa (in the intrinsic pathway) or by factor VIIa (in the extrinsic pathway). FXa is often characterized as a hemostatic agent and induces formation of thrombin during thrombosis. Thrombin activates tumor cell adhesion to platelets and endothelial cells and subsequently enhances tumor cell metastasis and angiogenesis (35). Jiang et al. found that adding the TF–FVIIa–FXa complex or FXa alone to the lower chamber promoted MCF-7 cell migration from the upper chamber toward the lower chamber (36, 37). In this study, we found a positive correlation between FX and TAM infiltration in human GBMs and GL261 cells intracranial xenograft tumors, and first discovered a novel function of FX in the chemotaxis of TAMs, which recruited macrophages and supported M2 subtype macrophage polarization to accelerate GBM growth *in vivo*, but did not affect GBM cell proliferation and invasion *in vitro*.

M1 TAMs play a role in antitumor immune responses, while M2 subtype TAMs are now widely regarded as immunosuppressive cells with a tumor-supportive role (38). CD11c is highly expressed in M1 subtype macrophages, and CD11c^HI^ cells were more capable of protein antigen processing and associated with APC function (39). By contrast, CD163 is highly expressed in M2 subtype macrophages. CD163 binds hemoglobin–haptoglobin (Hb–Hp) complexes, which leads to endocytosis and degradation of Hb–Hp by heme-oxygenase enzymes, resulting in an anti-inflammatory response (40). Our studies confirmed that FX was secreted to the tumor microenvironment and influenced macrophage polarization to the M2 subtype. We also found that FX secreted to GBM cell supernatants decreased THP-1 cell M1 subtype marker (IL-1β, IL-12α, CXCL9, IL-12β, and CXCL10) expression, while increasing M2 subtype markers (LYVE1, MRC1, STAB1, and SerpinB2) expression. Moreover, FX increased the proportion of M2 subtype (CD11b^+^CD206^+^) but decreased M1 subtype (CD11b^+^CD80^+^) macrophages. IL-1β is the most studied pro-inflammatory cytokine in the IL-1 family and is crucial for inflammation and tissue damage (41). M1 macrophages highly express chemokines such as CXCL9 and CXCL10, which attract Th1 cells (42). IL-12 comprises p35 (encoded by IL-12α) and p40 (encoded by IL-12β) chains and is considered as a pro-inflammatory molecule, which principally activates natural killer cells and induces naïve CD4^+^ T lymphocytes to differentiate into Th1 effector cells (43). These molecules decreasing with the stimulation of FX suggested that FX may inhibit M1 subtype macrophage polarization to protect tumor cells from immune cells. By contrast, HMOX-1 has been recognized as having immunomodulatory and anti-inflammatory properties, which drive the phenotypic shift to M2 macrophages (44). ARG1 depleted l-arginine by metabolizing it to urea and l-ornithine. l-arginine is necessary for T cell function, and its depletion suppresses T cell activity (45). MRC1 is an innate pattern-recognition receptor and has functions in clearing allergens and limiting allergic inflammation (46). SerpinB2 represents an immune-regulated factor that is critical for macrophage survival (47). The increase in molecules increased with FX stimulation indicated that FX may promote macrophages toward the M2 subtype to establish an immunosuppressive environment that is beneficial for tumor growth.

Previous studies have suggested that FX bound to the αMβ2 integrin subunit CD11b on activated monocytes, which was responsible for the conversion of prothrombin to thrombin (48). Integrin was also involved in mediating cell adhesion and migration. Blocking integrin α1β1 or knocking out integrin subunit alpha 1 increased macrophage exit from inflamed skin, suggesting that integrin is critical for controlling macrophage recruitment (49). Our results further confirmed that the GBM cell-secreted FX that mediates TAM recruitment and polarization may *via* CD11b. Blocking of integrin αM subunits with the CD11b antibody prevented macrophage recruitment and M2 subtype macrophage polarization induced by FX.

Integrin has been demonstrated to mediate cell proliferation, differentiation, and migration *via* the ERK pathway (50). The clustering of integrin αMβ2 inhibits the apoptosis of human neutrophils by activating ERK and AKT (51). Integrin α2β1 promotes T cell migration *via* activation of the ERK/Mcl-1 and p38 MAPK pathways (52). In macrophages, phosphorylation and activation of MAPK have been implicated in regulating macrophage polarization (53). The activation and proliferation of macrophages require ERK1/2 phosphorylation, and sustained activation of MAPK phosphorylation increased the expression of TNF-α, IL-6, and IL-10 (54). Hypoxia promotes M2 subtype polarization *via* the activation of ERK and enhances metastasis in non-small cell lung cancer (55). ROS generation induces ERK activation, resulting in macrophage polarization to the M2 subtype (56). Fortunately, in our study, we first confirmed FX bound to ERK1/2 in the cytoplasm, and more importantly, GBM cells secreting FX promoted the phosphorylation and activation of ERK1/2, resulting in macrophage M2 subtype polarization. In addition, activation of the PI3K/AKT pathway is critical for suppressing pro-inflammatory responses, while promoting anti-inflammatory responses in macrophages (57). The PI3K–AKT and MEK1/2–ERK1/2 pathways often collaborate and cross talk with each other (58). The ERK inhibiter U0126 and β-adrenoceptor decrease ERK phosphorylation while increases AKT phosphorylation (59). Other studies showed that the PI3K inhibitor wortmannin reduced p-ERK, and the ERK inhibitor FR180204 also enhanced p-AKT (60). AKT activation is required for M2 activation by the upregulation of M2 genes and several molecules, such as TGF-β and IL-10 (61). TIPE2 promotes M2 macrophage polarization *via* activation of the AKT signaling pathway (62). In our study, FX secreted by GBM cells and increased p-AKT in macrophages to promote M2 macrophage polarization. But FX did not interact with AKT in GBM cells. Interestingly, inhibition of p-ERK1/2 and p-AKT eliminated the effects of FX on M2 macrophage polarization. Combined with the results of the CD11b antibody on macrophage polarization, we proposed that FX secreted from GBM cells to the tumor environment recruited macrophages by interacting with CD11b on the surfaces of macrophages. Furthermore, FX bound to CD11b to promote phosphorylation and activation of ERK1/2 and AKT, resulting in M2 macrophage polarization. It is well known that the MAPK/ERK and PI3K/AKT pathways are reportedly associated with cell proliferation, differentiation, migration, senescence, and apoptosis (63). In our study, we found that FX bound to ERK1/2 and decreased p-ERK1/2 in GBM cells. These results contradicted with that FX did not influence the proliferation and invasion of GMB cells. Other molecule may involved in FX-mediated cell proliferation and further studies needed to prove.

miR-338-3p is reported to function as a tumor suppressor gene in various cancer, including hepatocellular carcinoma, neuroblastoma, ovarian cancer, gastric cancer, and colorectal cancer (64–66). In this study, we found that miR-338-3p targeted FX by binding to its 3′UTR in GBM cells. Our recent study showed that the lncRNA CASC2c directly bound miR-101 and influenced miR-101 to mature, and CASC2c acted as a competing endogenous RNA (ceRNA) of miR-101 to competitively regulate CPEB1 and promote the malignant growth of astrocytoma (67, 68). In this study, we first confirmed that in GBM cells, CASC2c repressed both miR-338-3p and FX. miR-338-3p bound CASC2c and inhibited CASC2c expression; on the other hand, CASC2c was a target gene of miR-338-3p and was repressed by miR-338-3p. Therefore, CASC2c and miR-338-3p formed a mutually inhibitory complex. However, CASC2c did not function as a ceRNA of miR-388-3p to competitively regulate FX expression. We found that CASC2c, along with miR-388-3p, inhibited FX expression. Yuan reported that the lncRNA ATB bound to IL-11 mRNA and increased IL-11 mRNA stability and secretion in hepatocellular carcinoma (69). Therefore, we proposed that CASC2c may bind FX mRNA directly and result in FX mRNA degradation. The mechanism needs to be studied further. In addition, both miR-338-3p and CASC2c in GBM cells repressed the migration of macrophages by inhibiting FX expression. CASC2c could influence macrophage polarization, but miR-338-3p could not. miR338-3p can target many proteins such as MMP2, MMP9 (70), BDNF (71), Sphk2 (72), and IRS2 (73). BDNF enriched in glioma environment stimulated the production of IL-15 in CD11b^+^ macrophages cells and altered macrophage plasticity (74). Sphk2 was demonstrated as a novel driver of the pro-inflammatory macrophage phenotype (75). IRS2 negatively regulates YM1 protein induction in macrophages and negatively regulated alternative macrophage activation (76). Therefore, although miR338-3p targets FX, miR338-3p did not influence macrophage polarization. In conclusion, CASC2c repressed FX expression synergistically with miR-338-3p and CASC2c regulated macrophage polarization.

**Figure 8:**
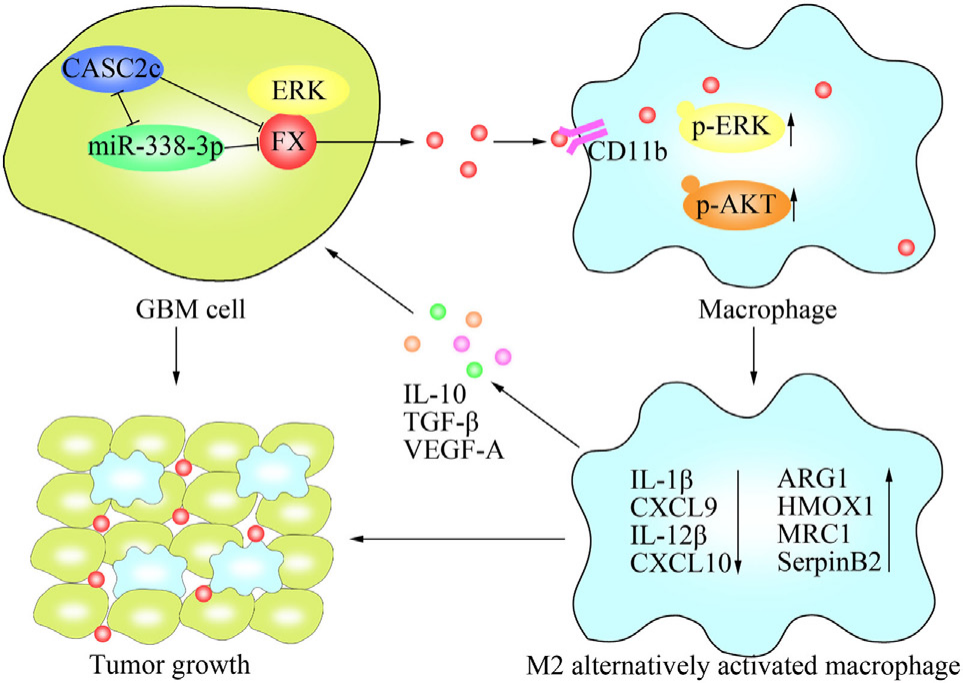
Schematic model of the molecular mechanisms representing the function of factor X (FX) in glioblastoma multiforme (GBM) tumor growth. FX secreted by GBM cells to the tumor microenvironment and recruited macrophages to the tumor *via* interactions with CD11b on the surfaces of macrophages. FX phosphorylated and activated extracellular signal-related kinase (ERK)1/2 and AKT in macrophages, leading to M2 subtype macrophage polarization, which facilitated GBM tumor growth. In GBM cells, miR-338-3p and lncRNA CASC2c inhibited the expression of FX. lncRNA CASC2c also interacted with miR-338-3p and reciprocally repressed it. FX interacted with ERK1/2 and decreased p-ERK1/2.

In summary, this study demonstrated a novel role of FX in GBM growth (Figure 8). FX was highly expressed in GBM cells, secreted to the tumor microenvironment and functioned as a potent chemokine in the chemoattraction of TAMs. FX recruited macrophages to promote tumor growth by interacting with CD11b on the surfaces of macrophages. FX promoted M2 subtype polarization by activating ERK1/2 and AKT in macrophages. In GBM cells, the lncRNA CASC2c interacted with and reciprocally repressed miR-338-3p; both CASC2c and miR-388-3p bound to FX, commonly inhibited the expression of FX. Our finding of TAM recruitment by FX may offer therapeutic potential to improve GBM therapy in patients.

## Ethics Statement

This study was carried out in accordance with the recommendations of the Joint Ethics Committee of the Central South University Health Authority with written informed consent from all subjects. All animal experiments were performed in accordance with the guidelines for the care of laboratory animals and the Animal Care and Use Committee of Central South University. The protocol was approved by the Joint Ethics Committee of the Central South University Health Authority.

## Author Contributions

YZ mainly performed the project and wrote the manuscript. JF, HF, CL, and ZY assisted with the animal experiments. YS and XS assisted with approving the final version of the manuscript. PL, CZ, YL, and TL assisted with the cell experiments and the data analysis. QiangL and QingL prepared the clinical samples. MW and GL developed the experimental design and revised the manuscript. All the authors approved the final manuscript.

## Conflict of Interest Statement

The authors declare that the research was conducted in the absence of any commercial or financial relationships that could be construed as a potential conflict of interest.

## Footnotes

^1^http://www.targetscan.org (Accessed: October 3, 2017).

^2^http://www.mircode.org (Accessed: October 3, 2017).

## Supplementary Material

Figure S1 Factor X (FX) influenced tumor growth *in vivo* but not *in vitro*. **(A)** Confocal microscopy showed GFP-FX localized to the trans-Golgi network and vesicles. **(B)** FX expression was increased in GL261-FX cells shown by Western blotting. **(C)** The viability of GL261-CON and GL261-FX was measured by XTT assays.

Figure S2 Factor X (FX) interacted with extracellular signal-related kinase (ERK)1/2 and influenced p-ERK1/2 and p-AKT. **(A)** FX was speculated to interact with ERK1/2 predicted by Scansite 3.0. **(B)** The interaction of FX and AKT was measured by co-immunoprecipitation.

Figure S3 CASC2c and miR-338-3p regulated factor X (FX) expression. **(A)** FX was speculated to be a target of miR-338-3p by TargetScan (http://www.targetscan.org). **(B)** CASC2c was speculated to interact with miR-338-3p by miRcode (http://www.mircode.org). **(C)** Nucleotide resolution of miRNA-binding sites in FX and miR-338-3p. **(D)** Nucleotide sequence of miRNA-binding sites in CASC2c and miR-338-3p.
