# Corrigendum: Coagulation Factor X Regulated by CASC2c Recruited Macrophages and Induced M2 Polarization in Glioblastoma Multiforme

**DOI:** 10.3389/fimmu.2020.00934

**Published:** 2020-05-29

**Authors:** Yan Zhang, Jianbo Feng, Haijuan Fu, Changhong Liu, Zhibin Yu, Yingnan Sun, Xiaoling She, Peiyao Li, Chunhua Zhao, Yang Liu, Tao Liu, Qiang Liu, Qing Liu, Guiyuan Li, Minghua Wu

**Affiliations:** ^1^Hunan Provincial Tumor Hospital and the Affiliated Tumor Hospital of Xiangya Medical School, Central South University, Changsha, China; ^2^The Key Laboratory of Carcinogenesis of the Chinese Ministry of Health, The Key Laboratory of Carcinogenesis and Cancer Invasion of the Chinese Ministry of Education, Cancer Research Institute, Central South University, Changsha, China; ^3^The Second Xiangya Hospital, Central South University, Changsha, China; ^4^The Third Xiangya Hospital, Central South University, Changsha, China; ^5^The Xiangya Hospital, Central South University, Changsha, China

**Keywords:** tumor-associated macrophages, polarization, glioblastoma multiforme, extracellular signal-related kinase 1/2, AKT

In Figure 4G the authors mistakenly used the wrong image. The fully corrected Figure 4 appears below.

**Figure 4:**
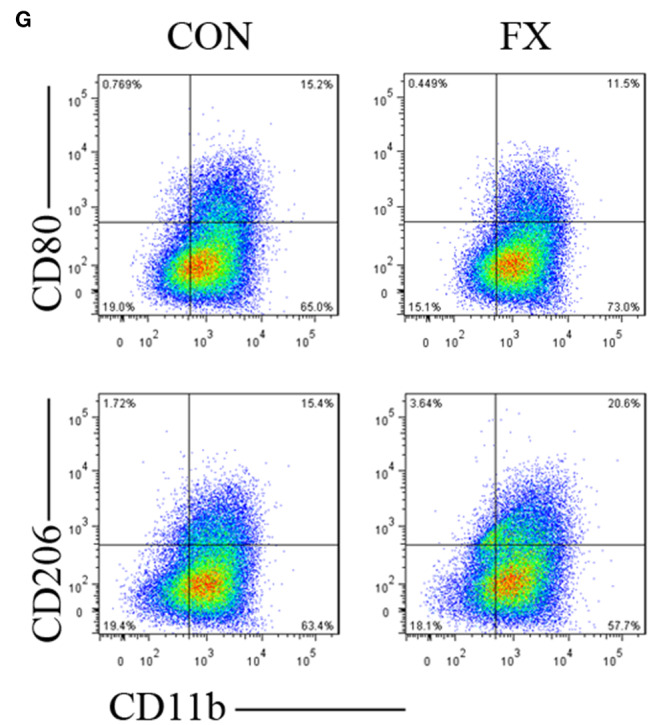
**(G)** The proportion of M1 macrophages decreased and M2 macrophages increased when THP-1 cells were treated with U251-FX cell supernatants compared with U251-CON.

The authors apologize for this error and state that this does not change the scientific conclusions of the article in any way. The original article has been updated.

