# Supplementary figures and images for: Coagulation Factor X Regulated by CASC2c Recruited Macrophages and Induced M2 Polarization in Glioblastoma Multiforme
Source: Front Immunol. 2018 Jul 6;9:1557. doi: 10.3389/fimmu.2018.01557 (PMC6043648; doi:10.3389/fimmu.2018.01557)

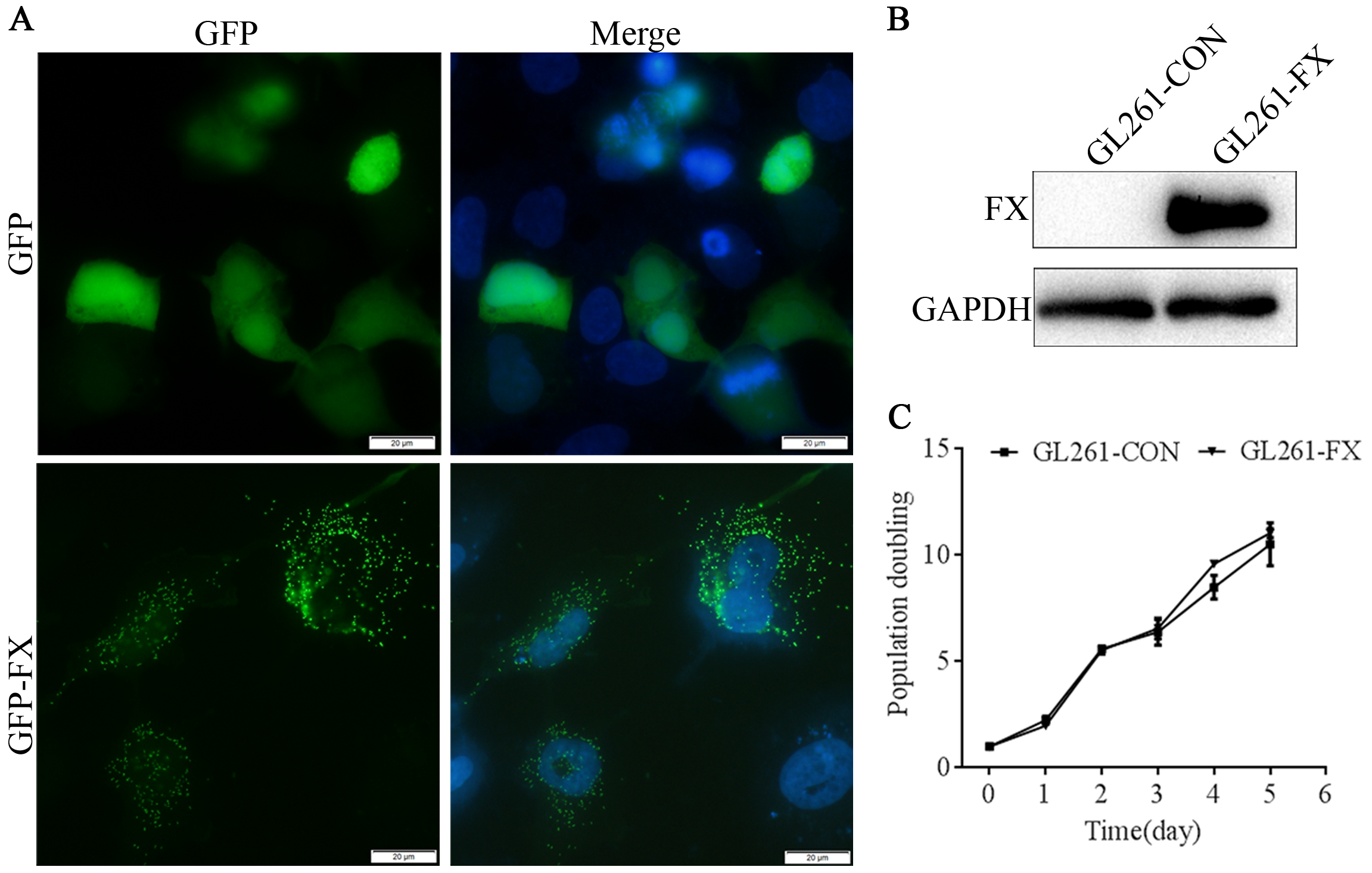

Supplement: Figure S1 — Factor X (FX) influenced tumor growth in vivo but not in vitro. (A) Confocal microscopy showed GFP-FX localized to the trans-Golgi network and vesicles. (B) FX expression was increased in GL261-FX cells shown by Western blotting. (C) The viability of GL261-CON and GL261-FX was measured by XTT assays. [file image_1.tif]

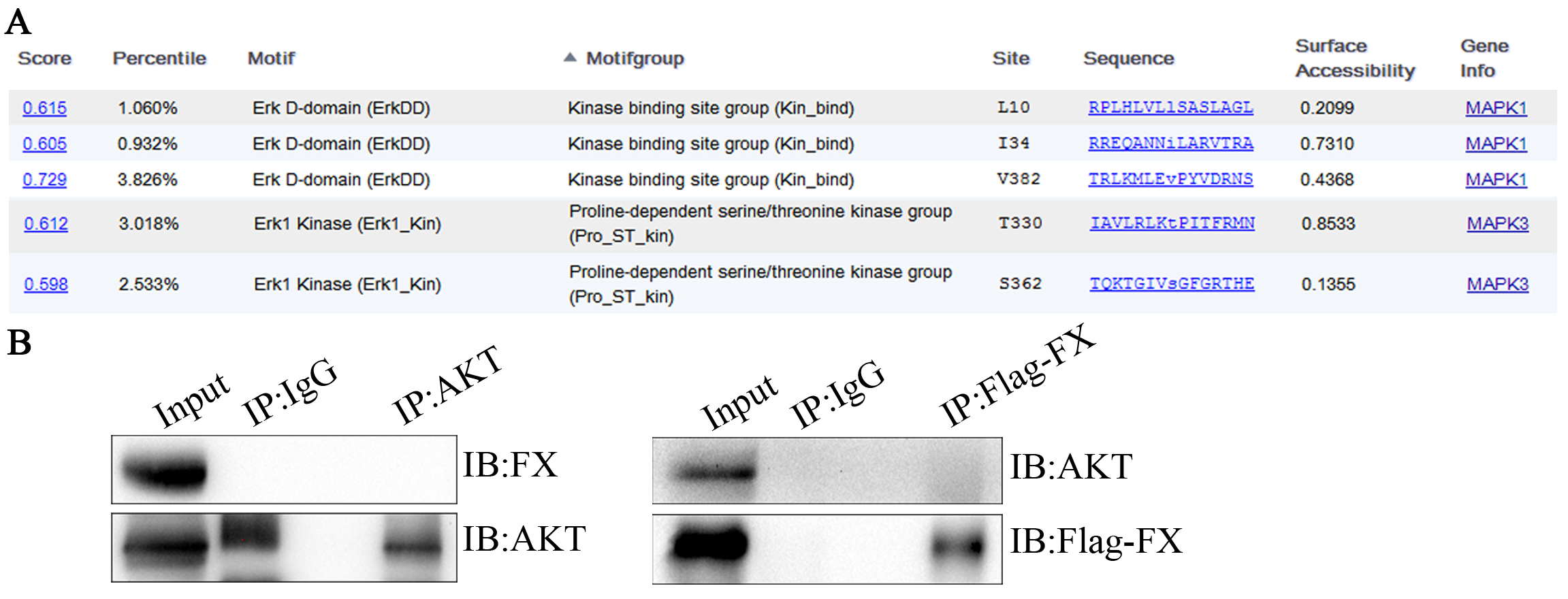

Supplement: Figure S2 — Factor X (FX) interacted with extracellular signal-related kinase (ERK)1/2 and influenced p-ERK1/2 and p-AKT. (A) FX was speculated to interact with ERK1/2 predicted by Scansite 3.0. (B) The interaction of FX and AKT was measured by co-immunoprecipitation. [file image_2.tif]

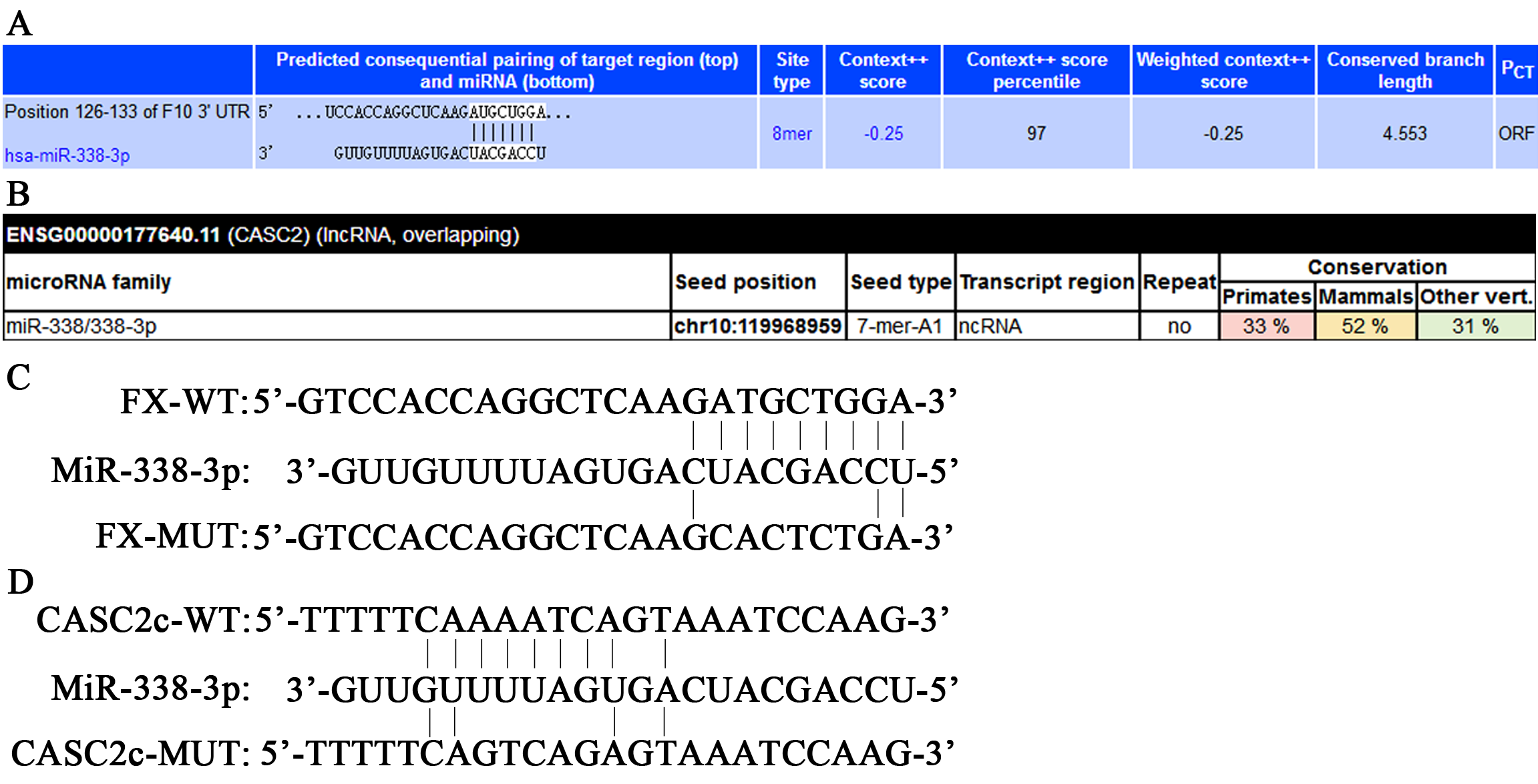

Supplement: Figure S3 — CASC2c and miR-338-3p regulated factor X (FX) expression. (A) FX was speculated to be a target of miR-338-3p by TargetScan (http://www.targetscan.org). (B) CASC2c was speculated to interact with miR-338-3p by miRcode (http://www.mircode.org). (C) Nucleotide resolution of miRNA-binding sites in FX and miR-338-3p. (D) Nucleotide sequence of miRNA-binding sites in CASC2c and miR-338-3p. [file image_3.tif]
