# Supplementary material for: Coagulation Factor X Regulated by CASC2c Recruited Macrophages and Induced M2 Polarization in Glioblastoma Multiforme
Source: Front Immunol. 2018 Jul 6;9:1557. doi: 10.3389/fimmu.2018.01557 (PMC6043648; doi:10.3389/fimmu.2018.01557)
Supplement: Supplementary file 4 [file table_1.docx]

**Table S1**. A list of primers used in the reactions for real-time PCR.

| Gene | Forward | Reverse |
| --- | --- | --- |
| GAPDH | ATCATCAGCAATGCCTCCT | CATCACGCCACAGTTTCC |
| FX | CTGGAACGCAGGAAGAGGT | GGCTGCCGTTAGGATGTAGA |
| CASC2c | TGGGTATTAGCCGACAGT | CTCCGTTGGTTATTGAAAGT |
| IL-1β | GATGGCTTATTACAGTGGCA | GTAGTGGTGGTCGGAGATT |
| IL-12α | GGCCCTGAATTTCAACAG | AATAGTCACTGCCCGAAT |
| CXCL9 | TTTCCTCTTGGGCATCAT | GGATAGTCCCTTGGTTGGT |
| IL-12β | TCAAACCTGACCCACCCA | GGACCTGAACGCAGAATGT |
| CXCL10 | TGCTGCCTTATCTTTCTG | CTCTTCTCACCCTTCTTTT |
| iNOS | ATGTACCCTCGGTTCTGCG | CTGAATGTGCTGTTTGCCTC |
| ARG1 | AAAAGCAAGCGAGCAGC | CACCCAGATGACTCCAAGA |
| HMOX1 | GCCAGCAACAAAGTGCAAGA | AGTGTAAGGACCCATCGGAGAA |
| LYVE1 | GGACCAAGTTGAAACAGCC | TCCAAATCAGGACACCCAC |
| MRC1 | TACCTGCGACAGTAAACGA | TGCTGGCTATAAGGGAAT |
| SerpinB2 | AGTGGACCAGCAAAGACAA | TCCGACATCCCTGAGAAA |
| STAB1 | GCCATCCGTCTCCTCCTTC | CCACCACCTTCGTGTTTGTT |
